# A clinical survey of mosaic single nucleotide variants in disease-causing genes detected by exome sequencing

**DOI:** 10.1186/s13073-019-0658-2

**Published:** 2019-07-26

**Authors:** Ye Cao, Mari J. Tokita, Edward S. Chen, Rajarshi Ghosh, Tiansheng Chen, Yanming Feng, Elizabeth Gorman, Federica Gibellini, Patricia A. Ward, Alicia Braxton, Xia Wang, Linyan Meng, Rui Xiao, Weimin Bi, Fan Xia, Christine M. Eng, Yaping Yang, Tomasz Gambin, Chad Shaw, Pengfei Liu, Pawel Stankiewicz

**Affiliations:** 1 Department of Molecular and Human Genetics, Baylor College of Medicine, One Baylor Plaza, Houston, TX 77030-3411 USA; 2 Baylor Genetics, Houston, TX USA; 3 Department of Obstetrics and Gynecology, The Chinese University of Hong Kong, Hong Kong SAR, China; 4 Department of Medical Genetics, Institute of Mother and Child, Warsaw, Poland; 5 Institute of Computer Science, Warsaw University of Technology, Warsaw, Poland; 6 Department of Statistics, Rice University, Houston, TX USA

**Keywords:** AOH, *CACNA1A*, CpG site, Somatic mosaicism, Genotype-phenotype correlation, PI3K-AKT-mTOR pathway, RASopathies, UPD

## Abstract

**Background:**

Although mosaic variation has been known to cause disease for decades, high-throughput sequencing technologies with the analytical sensitivity to consistently detect variants at reduced allelic fractions have only recently emerged as routine clinical diagnostic tests. To date, few systematic analyses of mosaic variants detected by diagnostic exome sequencing for diverse clinical indications have been performed.

**Methods:**

To investigate the frequency, type, allelic fraction, and phenotypic consequences of clinically relevant somatic mosaic single nucleotide variants (SNVs) and characteristics of the corresponding genes, we retrospectively queried reported mosaic variants from a cohort of ~ 12,000 samples submitted for clinical exome sequencing (ES) at Baylor Genetics.

**Results:**

We found 120 mosaic variants involving 107 genes, including 80 mosaic SNVs in proband samples and 40 in parental/grandparental samples. Average mosaic alternate allele fraction (AAF) detected in autosomes and in X-linked disease genes in females was 18.2% compared with 34.8% in X-linked disease genes in males. Of these mosaic variants, 74 variants (61.7%) were classified as pathogenic or likely pathogenic and 46 (38.3%) as variants of uncertain significance. Mosaic variants occurred in disease genes associated with autosomal dominant (AD) or AD/autosomal recessive (AR) (67/120, 55.8%), X-linked (33/120, 27.5%), AD/somatic (10/120, 8.3%), and AR (8/120, 6.7%) inheritance. Of note, 1.7% (2/120) of variants were found in genes in which only somatic events have been described. Nine genes had recurrent mosaic events in unrelated individuals which accounted for 18.3% (22/120) of all detected mosaic variants in this study. The proband group was enriched for mosaicism affecting Ras signaling pathway genes.

**Conclusions:**

In sum, an estimated 1.5% of all molecular diagnoses made in this cohort could be attributed to a mosaic variant detected in the proband, while parental mosaicism was identified in 0.3% of families analyzed. As ES design favors breadth over depth of coverage, this estimate of the prevalence of mosaic variants likely represents an underestimate of the total number of clinically relevant mosaic variants in our cohort.

**Electronic supplementary material:**

The online version of this article (10.1186/s13073-019-0658-2) contains supplementary material, which is available to authorized users.

## Background

Mosaicism is defined by the presence of different genotypic variants among cells of an individual that are derived from the same zygote [1]. Depending on the timing of mutation acquisition, mosaicism may be restricted to the germline (gonadal mosaicism) or non-germ cell tissues (somatic mosaicism) or may involve both (gonosomal mosaicism) [2]. It is estimated that three base substitution mutations arise per cell division in early human embryogenesis [3]. Postzygotic mutations dynamically accumulate and/or are negatively selected during the developmental process [4, 5], rendering each individual a complex mosaic of multiple genetically unique cell lines [1, 4].

Somatic mutations have been well known for their critical role in tumorigenesis [6] and overgrowth syndromes [5]. Mosaic variation has been reported also in asymptomatic individuals. In healthy donors, mutant allele fractions within organ samples ranged from 1.0 to 29.7% [7]. Mosaic variants may be clinically silent for several possible reasons: (1) the mutation is functionally inconsequential, (2) it is restricted to tissues not pertinent to the gene in which the mutation has arisen, (3) it may have occurred after a critical time frame for gene function, or (4) the mutation may be so disadvantageous that selective pressures favor survival and proliferation of cells carrying the reference allele.

Clinically relevant mosaicism is easily recognizable when cutaneous manifestations are present as with segmental neurofibromatosis or McCune-Albright syndrome [8]. However, in the absence of overt skin findings, recognizing underlying mosaicism may present a clinical challenge, particularly when the expressed phenotype deviates substantially from what has been reported in patients with non-mosaic variation. As patients with atypical phenotypes are often referred for exome sequencing (ES), an assessment of the performance of ES for detecting mosaic variation is warranted. Previous studies have evaluated the frequency and type of mosaic variation detectable by ES in specific disease populations, including neurodevelopmental disorders [9], autism [10, 11], and congenital heart disease [12]. However, few systematic analyses of mosaic variants detected by diagnostic ES for diverse clinical indications have been performed [13].

To address this gap in the literature and to lay a framework for additional studies of mosaicism in clinically relevant genes, we present a retrospective review of all reported mosaic variants detected in nearly 12,000 consecutive patients referred for diagnostic ES at Baylor Genetics (BG).

## Methods

### Study cohort

Laboratory reports for 11,992 consecutive unrelated patients referred for ES were queried to ascertain all clinically relevant mosaic variants reported between Nov 2011 and Aug 2018. Exome analyses were performed as trio ES in 19.8% (*n* = 2373) and proband-only ES in 80.2% (*n* = 9619) of cases. One hundred twenty clinical reports with mosaic variants were analyzed for this study; this included 30 cases (25%) analyzed by trio ES and 90 cases (75%) by proband-only ES. Only mosaic variants detected in DNA samples from peripheral blood were analyzed.

### Exome sequencing and analysis

ES was performed at BG laboratories as previously described [14, 15] (Additional file 1: Supplementary Methods). The validated ES protocol achieves a mean coverage of 130× with over 95% of targeted regions, including coding and untranslated exons, reaching a minimum coverage of 20×. All samples were concurrently analyzed by the HumanOmni1-Quad or HumanExome-12 v1 array (Illumina) for sample identity confirmation and to screen for copy-number variants and regions of homozygosity. Variant classification was performed in accordance with the American College of Medical Genetics and Genomics (ACMG) and Association for Molecular Pathology (AMP) guidelines for variant interpretation [16]. Mosaic variants of uncertain significance in our cohort that were reported prior to the publication of the ACMG/AMP guidelines were reassessed and classified according to the updated criteria. Common SNPs were filtered out from the analysis.

### Mosaic variants reporting/selection criteria

1. Alternate allele fraction (AAF) (mosaic variant reads/total reads) was calculated for each mosaic variant using the data generated by exome sequencing or PCR amplicon-based next-generation sequencing (NGS). For autosomal variants and X-linked variants in females, a variant was considered possibly mosaic if the AAF was less than 36% or greater than 64% by NGS analysis (Additional file 1: Supplementary Methods), while AAF higher than 10% was used as a threshold to identify mosaic variants in X-linked genes in males.
2. Mosaic variants detected by ES were orthogonally confirmed by Sanger sequencing. For mosaic variants ascertained by Sanger sequencing, a substantial and consistent reduction in the electropherogram peak height for the variant allele generated by the Mutation Quantifier function of the Mutation Surveyor software (SoftGenetics, State College, PA, USA) was deemed consistent with mosaicism. Mosaicism detected by Sanger sequencing was also confirmed by subsequent PCR amplicon-based NGS.
3. Only clinically reported mosaic variants were included in the analysis. Mosaic variants detected in disease genes not related to patient phenotype or in candidate disease genes and/or genes of uncertain significance were excluded from the analysis.
4. Mosaic variants detected in non-blood tissues were excluded from the study.

### NGS amplicon sequencing

PCR primers targeting mosaic variants were designed using “Primer 3” and synthesized by Sigma Genosys, Woodlands, TX, USA. For each sample, 40 ng of genomic DNA was amplified using Roche’s FastStart kit and/or GC-Rich PCR System for PCR. For *SLC6A8* and *TUBB* (genes with significantly homology to other regions of the genome), long-range PCR (TaKaRa long range PCR kit) followed by nested PCR was used. Amplicon size was checked by gel electrophoresis. PCR products were treated with Exonuclease-Shrimp Alkaline Phosphatase (New England’s BioLabs), and the SPRI bead purified products (Beckman and Coulter Inc. Brea, CA, USA) were used for bar-coding using Illumina compatible index adapters (Sigma Genosys, Woodlands, TX, USA). Barcoded samples were quantified by Qubit (Invitrogen, Life Technologies Corporation, Eugene, OR, USA) and sequenced using the Illumina HiSeq 2500 sequencing system with 100-bp paired-end reads (Illumina, San Diego, CA, USA).

### Computational analyses

To better assess the somatic mosaicism burden in ES data, we performed additional computational analyses of AAF distribution for heterozygous single nucleotide variants (SNVs) in 900 ES trios and simulation experiments for evaluating the effect of potential alignment biases.

## Results

**Fig. 1:**
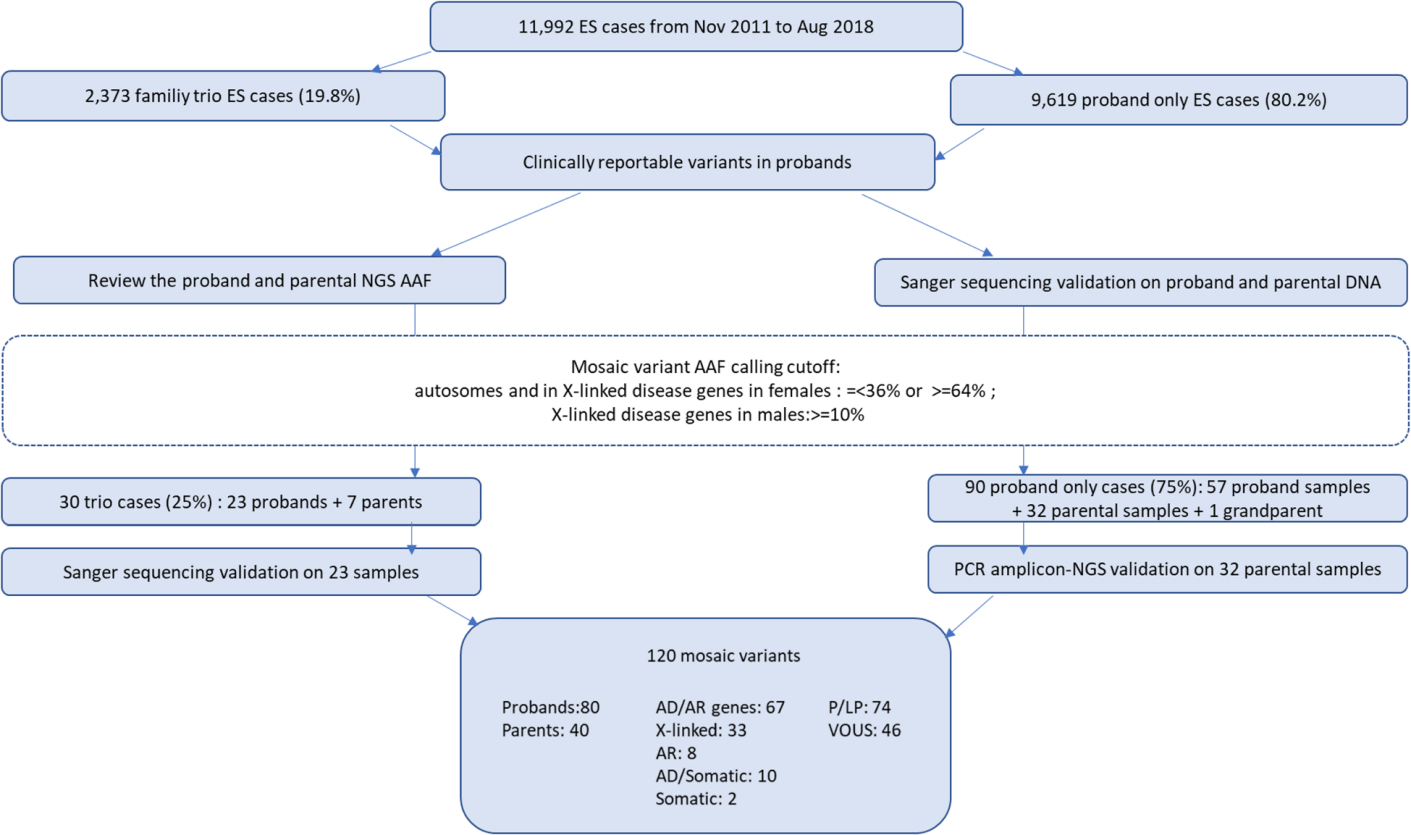
Overview of the SNV selection strategy

A total of 120 reported mosaic variants in 107 disease genes were detected in this cohort. Eighty-seven variants were detected by ES and 82 were confirmed by Sanger sequencing (Tables 1 and 2, Fig. 1), whereas 33 mosaic variants (in parental samples) were initially detected by Sanger sequencing. Thirty-two of 33 mosaic variants detected by Sanger sequencing were further validated using PCR amplicon-based NGS analysis (Table 2). For the 87 variants detected by ES, the average coverage at the site of the variant was approximately 202× (range 24–854×) while the average coverage of 32 variants assessed by amplicon-based NGS exceeded 10,000×. Average AAF of variants detected on autosomal chromosomes and in X-linked disease genes in females was 18.2% ± 9.5% (range 3.1–79.7%) compared with 34.8% ± 25.1% (range 10.0–85.0%) for X-linked disease gene variants detected in males. The AAF calculated based on the NGS data was significantly correlated (Spearman rho = 0.93, *p* = 0) with that quantified by Sanger sequencing (Additional file 2: Figure S1).

**Table 1:** The 80 mosaic variants detected in the probands

	Patient	Gene	Refseq ID	Mosaic variants	Category	AAF
AD	1F	*ARID1A*	NM_006015	c.2914delG (p.D972fs)	Path	17.5%
	2M	*ARID2*	NM_152641	c.4741C>T (p.P1581S)	VOUS	20.0%
	3M	*ASXL1*	NM_015338	c.2083_2084delCA (p.Q695fs)	Path	28.7%
	4M	*COL12A1*	NM_004370	c.533G>T (p.R178I)	VOUS	16.5%
	5F	***CREBBP***	NM_004380	c.5991delC (p.V1998fs)	Path	25.0%
	6M	***CREBBP***	NM_004380	c.1447C>T (p.R483*)	Path	23.3%
	7M	***DNM1***	NM_004408	c.415G>C (p.G139R)	LP	24.7%
	8M	*DNMT3A*	NM_175629	c.2260C>T(p.L754F)	LP	18.0%
	9M	*DYNC1H1*	NM_001376	c.5497G>A (p.A1833T)	VOUS	24.2%
	10M	*EEF1A2*	NM_001958	c.796C>T(p.R266W)	Path	32.0%
	11F	*ELN*	NM_001081755	c.1711G>A (p.A571T)	VOUS	22.8%
	12U	*ENG*	NM_000118	c.67+2T>G	LP	11.8%
	13F	*EP300*	NM_001429	c.2660C>T(p.T887I)	LP	12.0%
	14F	*GABRB2*	NM_000813	c.664G>T (p.V222F)	LP	20.8%
	15F	*GJA1*	NM_000165	c.433G>A(p.V145M)	VOUS	16.0%
	16F	*HNRNPK*	NM_002140	c.1003G>A (p.G335S)	VOUS	17.8%
	17M	*IDH2*	NM_002168	c.419G>A (p.R140Q)	Path	19.6%
	18M	*KANSL1*	NM_001193466	c.868C>T (p.R290*)	VOUS	11.6%
	19M	*KCNT1*	NM_020822	c.1421G>A (p.R474H)	Path	29.0%
	20F	*KIF1B*	NM_015074	c.2710G>A (p.E904K)	VOUS	17.3%
	21F	*KMT2A*	NM_001197104	c.3581G>A (p.C1194Y)	LP	36.0%
	22M	***KMT2D***	NM_003482	c.10938_10939delinsT (p.P3647fs)	Path	27.8%
	23M	***KMT2D***	NM_003482	c.8506C>T (p.R2836C)	VOUS	10.4%
	24F	*NALCN*	NM_052867	c.1783G>T (p.V595F)	LP	25.8%
	25M	*NF1*	NM_001042492	c.5907_5908delAA (p.R1970fs)	Path	10.4%
	26F	*NF2*	NM_000268	c.810+1G>T(N/A)	Path	15.0%
	27F	*NLRC4*	NM_021209	c.512C>T (p.S171F)	LP	25.3%
	28M	*NOTCH2*	NM_024408	c.118A>G (p.M40V)	VOUS	29.0%
	29F	***PIK3CA***	NM_006218	c.1359_1361delAGA(p.E453del)	Path	15.0%
	30M	*POLG*	NM_002693	c.2557C>T (p.R853W)	VOUS	10.4%
	31F	*PTPN11*	NM_002834	c.1403C>T(p.T468M)	LP	17.0%
	32M	*SNTG1*	NM_018967	c.814A>T (p.K272*)	VOUS	12.8%
	33F	*SYNGAP1*	NM_006772	c.1630C>T(p.R544*)	Path	11.0%
	34M	*TRAF7*	NM_032271	c.1111C>G (p.R371G)	VOUS	13.3%
	35M	*TWIST2*	NM_057179	c.223G>C(p.E75Q)	Path	24.0%
	36F	*ROBO1*	NM_002941	c.3055T>G(p.Y1019D)	LP	20.0%
AD/AR	37F	*ACTA1*	NM_001100	c.1003C>T (p.P335S)	VOUS	14.4%
	38F	*COL6A3*	NM_004369	c.3932A>T(p.N1311I)	VOUS	25.0%
	39F	*SLC25A4*	NM_001151	c.706C>T (p.R236C)	VOUS	15.2%
AD/somatic	40U	*BRAF*	NM_004333	c.1786G>C (p.G596R)	VOUS	15.6%
	41M	*KRAS*	NM_004985	c.355G>A (p.D119N)	VOUS	20.8%
	42M	*WT1*	NM_024426	c.865_867delinsAA (p.Y289fs)	Path	34.6%
	43F	*HRAS*	NM_005343	c.38G>A (p.G13D)	Path	19.0%
	44M	***MTOR***	NM_004958	c.7255G>A (p.E2419K)	LP	22.3%
	45M	***MTOR***	NM_004958	c.7247C>A (p.A2416D)	LP	16.0%
	46M	***MTOR***	NM_004958	c.5930C>T (p.T1977I)	Path	21.5%
	47M	***PIK3CA***	NM_006218	c.1030G>A (p.V344M)	LP	24.4%
	48F	***PIK3CA***	NM_006218	c.1093G>A (p.E365K)	Path	12.7%
AR	49M	*ADGRV1*	NM_032119	c.16368G>T (p.K5456N)	VOUS	23.7%
	50M	*ALG6*	NM_013339	c.52C>T (p.R18*)	Path	17.9%
	51F	*COX15*	NM_004376	c.1129A>T (p.K377*)	Path	12.1%
	52F	*CWF19L1*	NM_018294	c.70delA (p.R24fs)	Path	14.0%
	53M	*GNPTG*	NM_032520	c.376G>A (p.G126S)	VOUS	18.7%
	54F	*ZMPSTE24*	NM_005857	c.1077dupT (p.L362fs)	VOUS	79.7%
Somatic	55M	*IDH1*	NM_005896	c.395G>A (p.R132H)	LP	14.0%
	56M	*TET2*	NM_001127208	c.3961A>T (p.K1321*)	VOUS	30.2%
XL	57F	*ALG13*	NM_001099922	c.320A>G (p.N107S)	Path	10.9%
	58F	*CDKL5*	NM_003159	c.593G>A (p.G198D)	VOUS	11.6%
	59F	***DDX3X***	NM_001193416	c.573_575del (p.I191del)	LP	21.1%
	60F	***DDX3X***	NM_001193416	c.1805G>A (p.R602Q)	VOUS	13.9%
	61F	*HCFC1*	NM_005334	c.1004A>G (p.Y335C)	LP	16.4%
	62F	*NAA10*	NM_003491	c.247C>T (p.R83C)	Path	16.2%
	63F	*OPHN1*	NM_002547	c.1817C>T (p.S606F)	VOUS	18.2%
	64F	*SNX14*	NM_153816	c.1050T>A(p.F350L)	VOUS	18.0%
	65F	*ZC4H2*	NM_018684	c.199C>T (p.R67*)	LP	24.2%
	66M	*NEXMIF*	NM_001008537	c.862G>T(p.E288*)	Path	20.0%
	67M	*CASK*	NM_003688	c.913_914dupAA (p.G306fs)	Path	47.2%
	68M	*CLIC2*	NM_001289	c.255A>T(p.K85N)	VOUS	11.0%
	69M	*DMD*	NM_004006	c.583C>T (p.R195*)	Path	14.3%
	70M	***GRIA3***	NM_000828	c.1936T>C (p.S646P)	VOUS	61.9%
	71M	***GRIA3***	NM_000828	c.1981A>G (p.M661V)	LP	37.9%
	72M	*HUWE1*	NM_031407	c.8987G>A(p.R2996Q)	VOUS	13.0%
	73M	*KDM5C*	NM_004187	c.469T>A (p.Y157N)	LP	44.6%
	74M	*KDM6A*	NM_021140	c.2172_2173delAT(p.L725fs)	Path	11.0%
	75M	*L1CAM*	NM_000425	c.2357T>A(p.I786N)	LP	85.0%
	76M	*OTC*	NM_000531	c.1048C>T (p.Q350*)	Path	10.8%
	77M	*PCDH19*	NM_001184880	c.919G>A (p.E307K)	VOUS	75.9%
	78M	*PDHA1*	NM_000284	c.265G>A (p.G89S)	VOUS	17.1%
	79M	*TTN*	NM_133378	c.87881T>C(p.V29294A)	VOUS	10.0%
	80M	*UBA1*	NM_003334	c.1631G>A (p.R544Q)	VOUS	30.1%

*F* female, *M* male, *Path* pathogenic, *LP* likely pathogenic, *AAF* alternate allele fraction

Bold genes showed up more than one time

**Table 2:** The 40 mosaic variants detected in the parental or grandparental samples

	Patient ID	Gene	Refseq ID	Mosaic variants	Category	AAF
Detected in the mother						
AD	81 M-PGM	***DYRK1A***	NM_001396	c.41C>T (p.S14F)	VOUS	.
	82M-Mo	*ATP1A3*	NM_152296	c.410C>A (p.S137Y)	Path	14.9%
	83M-Mo	***CACNA1A***	NM_001127221	c.400-3C>T(N/A)	LP	27.7%
	84U-Mo	*COL4A1*	NM_001845	c.2879G>T (p.G960V)	LP	17.6%
	85F-Mo	*EPHA7*	NM_004440	c.595A>T (p.K199*)	VOUS	8.7%
	86F-Mo	*FGFR2*	NM_000141	c.289G>A (p.A97T)	VOUS	18.3%
	87F-Mo	*GARS*	NM_002047	c.815T>G (p.L272R)	VOUS	21.1%
	88M-Mo	*GH1*	NM_000515	c.291+2T>G	Path	11.8%
	89F-Mo	*GNAO1*	NM_020988	c.736G>C(p.E246Q)	LP	9.7%
	90F-Mo	*MPZ*	NM_000530	c.392A>C (p.N131T)	LP	13.2%
	91U-Mo*	*MYH3*	NM_002470	c.2015G>A (p.R672H)	Path	7.4%
	92M-Mo	*SCN1B*	NM_199037	c.794G>C (p.R265P)	VOUS	33.2%
	93M-Mo*	*TUBB*	NM_178014	c.860C>T (p.P287L)	LP	3.1%
AD/AR	94M-Mo	*TUBB3*	NM_006086	c.862G>C (p.E288Q)	LP	17.3%
	95M-Mo	*MAT1A*	NM_000429	c.896G>A (p.R299H)	VOUS	14.3%
AD/somatic	96M-Mo	*ZNF423*	NM_015069	c.2531G>A (p.G844E)	LP	25.3%
AR	97M-Mo	*FGFR1*	NM_023110	c.1982G>A (p.R661Q)	VOUS	20.4%
XL	98F-Mo	*FAT4*	NM_024582	c.8805C>A (p.Y2935*)	Path	9.1%
	99M-Mo	*ARX*	NM_139058	c.1003T>C (p.F335L)	VOUS	7.8%
	100M-Mo	*ATP7A*	NM_000052	c.3445C>T (p.Q1149*)	Path	6.5%
	101F-Mo	*ATRX*	NM_000489	c.477delA (p.K159fs)	Path	10.1%
	102M-Mo	*AVPR2*	NM_000054	c.335G>T (p.C112F)	LP	11.4%
	103M-Mo	*CUL4B*	NM_003588	c.2722C>T (p.Q908*)	VOUS	3.1%
	104M-Mo*	*SLC16A2*	NM_006517	c.590G>A (p.R197H)	Path	6.0%
	105M-Mo	*SLC6A8*	NM_005629	c.1697T>C (p.L566P)	VOUS	15.5%
Detected in the father						
AD	107F-Fa	*ADCY5*	NM_183357	c.3574C>T (p.R1192*)	VOUS	17.1%
	108M-Fa	*ARID1B*	NM_020732	c.6322C>T (p.Q2108*)	Path	6.8%
	109M-Fa	***CACNA1A***	NM_001127221	c.3533C>T (p.P1178L)	VOUS	29.5%
	110M-Fa	***CACNA1A***	NM_001127221	c.653C>T (p.S218L)	Path	15.7%
	111M-Fa*	*COL1A1*	NM_000088	c.3709_3716del (p.S1237fs)	Path	6.4%
	112F-Fa	***CREBBP***	NM_004380	c.5238_5239delinsT (p.L1747fs)	Path	33.2%
	113F-Fa	***DNM1***	NM_004408	c.709C>T (p.R237W)	Path	8.1%
	114M-Fa	***DYRK1A***	NM_001396	c.1162dupG (p.A388fs)	Path	17.6%
	115F-Fa*	*SATB2*	NM_015265	c.1174G>C (p.G392R)	LP	15.2%
	116F-Fa*	*SCN2A*	NM_021007	c.2562+1G>T	Path	24.6%
	117F-Fa	*SPTLC1*	NM_006415	c.1072G>C (p.E358Q)	LP	6.5%
	118F-Fa	*STXBP1*	NM_003165	c.704G>C (p.R235P)	LP	10.8%
AR	119F-Fa	*TRIO*	NM_007118	c.4505G>A (p.R1502Q)	LP	23.1%
XL	120F-Fa	*COL4A5*	NM_000495	c.2365A>C (p.T789P)	VOUS	67.8%

*F* female, *M* male, *PGM* paternal grandmother, *Mo* Mother, *Fa* Father, *Path* pathogenic, *LP* likely pathogenic, *AAF* alternate allele fraction

Bolded genes showed up more than one time. *AAF of this case were estimated by exome sequencing, the rest cases without * were performed with PCR-based amplicon-NGS

Mosaic variants occurred in genes associated with all types of inheritance, including autosomal dominant (AD) or AD/autosomal recessive (AR) (67/120, 55.8%), X-linked (33/120, 27.5%), AD/somatic (10/120, 8.3%), and AR (8/120, 6.7%) inheritance (Additional file 3: Table S1). Two of the 120 identified mosaic variants involved the *IDH1* (MIM 137800) and *TET2* (MIM 614286) genes in which only somatic events have been described. Nine genes, including *CACNA1A*, *CREBBP*, *MTOR*, and *PIK3CA* (*n* = 3 each), and *DDX3X*, *DNM1*, *DYRK1A*, *GRIA3*, and *KMT2D* (*n* = 2 each) harbored recurrent mosaic events in unrelated individuals. The observed mosaic variants included missense 67.5% (81/120), nonsense 14.1% (17/120), frameshift or in-frame del/dup 13.3% (16/120), and splice 5.0% (6/120) changes (Additional file 3: Table S2). Simulation experiments did not show potential alignment bias of different types of mutations (Additional file 2: Figure S2-S4). Of all single nucleotide substitution variants, 33.7% (35/104) involved CpG sites (Additional file 3: Table S2), and nucleotide C/G>T/A was the most common substitution change (Additional file 3: Table S3).

### Mosaic variants in probands

In proband samples, 80 mosaic variants were found in 72 genes in 33 female patients, 45 male patients, and two fetuses. The vast majority were reported in genes associated with AD (47.5%) and X-linked (30.0%) disorders. Mean AAF in proband samples was 32.6% ± 24.4% (*n* = 15) for X-linked variants in males and 20.2% ± 9.8% (*n* = 65) for autosomal variants and variants in X-linked disease genes in females (Table 1, Additional file 3: Table S4). For 65 of the 80 probands with mosaic variants, both parental samples were available for inheritance determination. Eight probands had only one parental sample available, and 7 probands had no parental samples available for analysis. The majority of mosaic variants detected in probands (63/65) were deemed de novo due to the absence of the variant in parental DNA by Sanger sequencing. Parental chromosome of origin could not be determined due to a lack of informative SNPs flanking the mosaic variants. In patient 55F, a c.1077dupT (p.L362fs) change in *ZMPSTE24* (an autosomal recessive disease gene) was found at an AAF of 80% due to suspected uniparental disomy (UPD) involving chromosome 1. In patient 52F, an inherited c.1129A>T (p.K377*) change in *COX15* (also an autosomal recessive disease gene) was found at an AAF of 12% due to suspected segmental UPD involving chromosome 10.

Of the mosaic variants detected in the proband samples, 58.8% (*n* = 47) were classified as pathogenic (P) or likely pathogenic (LP), and 41.3% (*n* = 33) as variants of uncertain significance (VOUS). For probands with a mosaic VOUS, 36.4% (12/33) were reported together with one or more non-mosaic P/LP mutations, including de novo or biallelic changes that could explain the core phenotype in four cases, and a heterozygous P/LP variant in an autosomal recessive disease gene in eight cases.

Genotype-phenotype analysis was performed for 47 patients with mosaic P/LP variants (Additional file 4) [17]. Eighty-three percent of the patients had core phenotypes that were consistent with what had been previously reported in association with heterozygous variants, with no evidence of disease attenuation related to the mosaic status of the variant. However, patient 43F carrying a c.38G>A (p.G13D) variant with an AAF 20.8% in *HRAS* had an apparently attenuated Costello syndrome phenotype, mirroring but less severe than typical for patients with germline mutations in this gene. Three patients had mosaic variants that, even if fully penetrant, would not have explained the full scope of the clinical presentation, including patient 12U with a c.67+2T>G variant in *ENG*; patient 69M with a c.583C>T (p.R195*) in *DMD*; and patient 79M with a c.87881T>C (p.V29294A) variant in *TTN*. We also found three patients with dual molecular diagnoses in whom a second non-mosaic pathogenic variant was considered contributory to the patient’s phenotype (patients 12U, 27F, and 35M). Two patients had multiple mosaic variants detected, including patient 3M who had 17 mosaic variants, only two of which were clinically reported and included in this analysis (see “Discussion”). Patient 12U had eight mosaic variants detected, but only one was found in a known disease-associated gene; the remaining mosaic variants were excluded from this analysis. In both cases, it was unclear whether the mosaic variants had contributed to the patient’s phenotype or if they were a consequence of an underlying predisposition to somatic mutation in the context of a pre-cancerous or cancerous state.

### Mosaic variants in parental samples

Forty mosaic variants in 37 genes were detected in 40 parental samples, including one variant detected in a grandparental sample (Table 2). Seven mosaic variants were identified by trio ES analysis whereas the remaining 33 variants were found by Sanger sequencing. Thirty-two of 33 mosaic variants detected by Sanger sequencing were confirmed by PCR-based amplicon NGS. The average AAF of variants detected in autosomal chromosomes and in X-linked disease genes in maternal samples was 14.6 ± 8.0% (Additional file 3: Table S4). One father (120F-Fa) had a mosaic variant with an AAF of 67.8% in the X-linked disease gene, *COL4A5*, which was detected as a heterozygous change in his daughter. 67.5% (27/40) of mosaic variants detected in parental samples were classified as P/LP in the proband. However, the majority of parents harboring mosaic variants were reported to be clinically unaffected. Only two parents with mosaic variants exhibited phenotypes related to the mosaic change. The father of patient 120F (120F-Fa) with a c.2365A>C (p.T789P) variant in *COL4A5* associated with X-linked Alport syndrome (MIM:301050), was reported to have a renal defect. The mother of patient 82M (82M-Mo) was reported to have seizures, muscle weakness, leg weakness, and a clumsy gait; she was found to have a mosaic c.410C>A (p.S137Y) variant in *ATP1A3* with an AAF of 14.9%. *ATP1A3* is associated with the autosomal dominant disorders, Dystonia 12 (DYT12) [MIM:128235] and cerebellar ataxia, areflexia, *pes cavus*, optic atrophy, and sensorineural hearing loss (CAPOS) [MIM:601338]. Interestingly, mosaic variants in the *CACNA1A* gene with AAFs ranging from 15.7 to 29.5% were exclusively detected in parental samples (*n* = 3). In contrast, mosaic variants in *MTOR* with comparable AAFs ranging from 16.0 to 32.0% were exclusively detected in proband samples.

## Discussion

Each cell division brings with it a risk of a new mutation. Mutations that occur after fertilization lead to the formation of distinct cell lineages or a state of genetic mosaicism. Depending on the functional consequence of the mutation, the timing of its acquisition, and its tissue distribution, the effect of a mosaic variant on patient phenotype can range from negligible to catastrophic. Although mosaic variation has been known to cause disease for decades, high-throughput sequencing technologies with the analytical sensitivity to consistently detect variants at reduced allelic fractions have only recently emerged as routine clinical diagnostic tests. Therefore, empirical studies of the frequency of mosaicism in large patient populations are only now being performed and published. The incidence of mosaic CNVs and aneuploidy found in patients referred for microarray testing has been estimated at 0.55–1% [18, 19]. Without additional verification studies, it is challenging in routine ES analyses to distinguish real somatic variants from apparently de novo heterozygous variants with highly skewed (lower than 0.36) AAF. Therefore, we have focused here only on clinically relevant SNVs. A systematic assessment of the rate of clinically relevant mosaic variant detection in large cohorts of individuals referred for ES with heterogeneous clinical presentations needs more investigations [13].

We endeavored to study the frequency, type, allelic fraction, and phenotypic consequences of reportable mosaic SNVs in a cohort of nearly 12,000 consecutive unrelated patients referred for clinical ES. A total of 120 mosaic variants in 107 established disease genes were detected and reported in either proband (*n* = 80) or parental (*n* = 39)/grandparental (*n* = 1) samples. Mosaic variation was considered definitely or possibly contributory to disease in approximately 1% of 11,992 subjects in this study. Assuming a molecular diagnosis was ascertained in 25% of patients in this cohort [14], an estimated 1.5% of all molecular diagnoses could be attributed to a mosaic variant detected in the proband samples. The fact that these estimates are low relative to other published cohorts was anticipated, as existing reports have studied mosaicism in specific genes [9, 20] or phenotypes [10, 11, 21], and/or have assessed the frequency of rare mosaic variants [11] but not specifically clinically reportable variants.

To assess the phenotypic effects of mosaicism in our cohort, we analyzed the provided clinical information and compared the phenotype of each patient to descriptions in the literature and/or in Online Mendelian Inheritance in Man (OMIM) of individuals with predominantly non-mosaic mutations. In the vast majority of probands with mosaic P/LP variants in AD/X-linked/somatic genes and no confounding factors (e.g., presence of multiple mosaic variants, underlying structural variation), the clinical presentation was not appreciably diminished in severity. In contrast, among parents with mosaic variants, only two (82M-Mo, 120F-Fa) were reported to have a phenotype that could be attributed to the identified mosaic mutation. Excluding mosaic variants detected in X-linked genes in males, a comparison of the AAF of mosaic variants in parental samples (14.6% ± 8.0%) relative to proband samples (20.0% ± 9.8%) showed that unaffected parents with mosaic variants have a significantly lower AAF (*p* = 0.004, *t*-test). It is intriguing that mosaic variants with ~ 5% lower AAFs can result in mild or absent phenotypes or can cause clinically significant manifestations. One explanation would be that the impact of any given postzygotic variant is likely to be dependent on the biological function of the gene and the distribution of the mutation in critical tissues. This notion is supported by the mosaic variants found in *MTOR*, *PIK3CA*, and *CACNA1A* in our study. Mosaic variants in *MTOR* and *PIK3CA* with AAFs ranging from 12.7 to 24.4% were detected in affected probands with Smith-Kingsmore syndrome [MIM: 616638], Cowden syndrome 5 [MIM: 615108], and/or megalencephaly-capillary malformation-polymicrogyria syndrome [MIM: 602501]. Conversely, mosaic variants in *CACNA1A* with similar AAFs ranging from 15.7 to 29.5% were all detected in asymptomatic parents. The contrasting severity of phenotypes seen in probands versus clinically unaffected parents highlights the challenge of predicting phenotypic outcomes based on genetic testing alone. It also raises the question of how variant mosaicism should be weighed in the course of variant classification given that both pathogenic and benign effects are possible depending on the clinical context in which the variant is detected.

Interestingly, recurrent mosaic variants in a subset of 9 genes: *MTOR*, *CREBBP*, *CACNA1A*, *DDX3X*, *DNM1*, *DYRK1A*, *GRIA3*, *KMT2D*, and *PIK3CA* accounted for 18.3% (22/120) of all detected mosaic variants in the analyzed cohort. Mosaic variants in several of these genes have been reported previously in the literature: *MTOR* [11], *CREBBP* [22], *CACNA1A* [23], *DNM1* [24], *KMT2D* [25], and *PIK3CA* [26]*.* In some cases, e.g., the *MTOR* and *PIK3CA* genes, somatic variants are the predominant or the only form of disease-causing mutation described in affected individuals. We have also noted that 10 (12.5%) of the 80 de novo mosaic variants detected in the proband samples were found in a gene associated with the Ras or PI3K-AKT-mTOR pathway, including one variant each in *BRAF*, *NF1*, *HRAS*, and *KRAS*, and three variants in *PIK3CA* and *MTOR*. Heterozygous variants in the same six genes were reported in less than 1% of the entire cohort, indicating that mosaic variation is disproportionately likely to affect this pathway. In fact, mosaic events in this pathway have been commonly observed [27]. The reason for enrichment of mosaicism in the Ras or PI3K-AKT-mTOR signaling pathway is unclear; possible explanations include (1) preferential expansion of hematologic clones with variants in these genes increasing the likelihood of mosaic variant detection, (2) high penetrance of mosaic variants in Ras pathway genes relative to other genes, and (3) a preponderance of intragenic mutation-prone residues.

The recognition that certain genes are more prone to pathogenic postzygotic mutation critically informs recurrence risk counseling and enables optimization of test development and data interpretation in the diagnostic lab setting. Panel-based tests targeting genes with recurrent mosaic variants should have sufficient depth of coverage and, to account for the risk of parental mosaicism, should include recommendations for parental testing. AAF filters are often utilized for comprehensive genomic assays such as exome and whole genome sequencing to exclude variants that are likely to represent sequencing artifact, a practice that can preclude detection of low-level mosaicism. Even with an average ES read depth of 130×, mosaic variants with AAF of less than 10% may be filtered out and excluded from review. For these methodologies, relaxing AAF filters for a defined subset of phenotypically relevant genes in which recurrent mosaic events are known to occur may help to optimize mosaic variant detection. Additionally, testing of tissues distant from the hematopoietic lineage (e.g., urine or hair follicles) could be performed to confirm mosaic status [7].

Adding to the complexity of mosaic variant interpretation, several patients in our cohort were found to harbor more than one mosaic variant. One patient (12U) with multiple congenital malformations was found to have compound heterozygous variants in *RAD51C*, a gene associated with Fanconi anemia [28], a mosaic VOUS in *ENG*, and seven additional mosaic variants in genes with no definitive disease association. Genomic instability resulting from spontaneous chromosome breakage is a hallmark of FA [29] and previous studies have shown an increased risk of mosaic copy-number and structural variants in affected individuals [30]. However, the impact of underlying FA on acquisition of somatic single nucleotide and small insertion/deletion variants has not been clearly elucidated. Therefore, although likely, the mosaic variants detected in this patient cannot be unequivocally attributed to the FA diagnosis. Multiple mosaic variants (*n* = 17) were also detected in patient 3M referred for ES with a history of malignant astrocytoma, myelodysplasia, and dysmorphic features. The mosaic mutations detected in this individual were likely related to the patient’s recent history of myelodysplastic syndrome. Although the phenomenon of mutation acquisition in pre-cancerous and cancerous states is not novel [31], multiple mosaic events stemming from malignancy can be an unexpected finding on assays like ES that are generally performed for the detection of germline, rather than somatic mutations. These findings are also challenging from the standpoint of clinical follow-up, as guidelines do not exist to direct management of incidentally ascertained cancer variants in individuals without a known malignancy.

Finally, we have noted that SNV mosaicism can also be explained by chromosomal abnormalities. Patient 52F with developmental delay and microcephaly was found to have a pathogenic variant in the *COX15* gene detected at an AAF of 12%. Analysis of the parental samples for the pathogenic change indicated that the father was heterozygous and the mother was negative for the variant. Due to the unexpectedly low AAF in the proband of the purportedly inherited *COX15* variant, review of the SNP array data was performed and the mosaic maternal uniparental disomy of distal chromosome 10q encompassing the *COX15* gene was found. In a second case, patient 55F with macrocephaly, dysmorphic features, and digital anomalies was found to have a mosaic pathogenic variant in *ZMPSTE24* at an AAF of 80%. The pathogenic variant was found to be heterozygous in the mother and negative in the father. Analysis of the SNP array data again revealed mosaic copy neutral AOH suspicious for UPD involving chromosome 1 and encompassing the *ZMPSTE24* gene, which presumably served as the “second hit” for the autosomal recessive disorder.

The many variables that complicate mosaic variant interpretation can also be leveraged in research studies to make inferences about variant pathogenicity and to provide insights into gene function. For example, from the observation that activating mutations in *GNAS* (associated with McCune-Albright syndrome, OMIM 174800) are detected only in the mosaic state, one can infer that constitutional activating mutations in this gene are incompatible with life [8, 32]. It is plausible that studies of affected individuals, including analyses of AAF by tissue type, would help to define key aspects of gene function, including after what critical developmental period the mutation must occur to ensure viability. For example, conditional *PIK3CA* activation in mouse cortex showed that abnormal mTOR activation in excitatory neurons and glia, but not interneurons, is sufficient for abnormal cortical overgrowth [33].

Although our cohort is comprised of nearly 12,000 families and we have detected and reported 120 mosaic mutations, only a minority of individuals were found to have mosaic variants in the same gene, which limits our ability to draw conclusions about gene function from analysis of mosaic variation in this cohort specifically. Moreover, causative mutations may be restricted to brain or other tissues that are not commonly studied sources of DNA [34]. As such, additional studies dedicated to assessing mosaicism including larger cohorts of affected and unaffected individuals will be necessary to accumulate the evidence needed to make broad conclusions about gene function based on mosaic variation in the population. Such studies may also allow the use of quantitative information, such as AAF, to predict clinical phenotype, particularly if multiple tissues can be analyzed. Finally, single-cell sequencing will permit a more accurate evaluation of the role of somatic mutations in neurodevelopmental disorders and during normal brain development [35].

## Conclusions

In summary, in our cohort of nearly 12,000 patients/families referred for clinical diagnostic ES, mosaic variants considered likely or definitively contributory to phenotype were detected in approximately 1.5% of probands in whom a molecular diagnosis was ascertained. Parental mosaicism was identified in 0.3% of families analyzed. We observed that certain genes, pathways, and even individuals were prone to mosaic variation and that SNV mosaicism can be an indication of underlying structural variation. Since clinical ES by design favors breadth over depth of coverage and only blood samples were analyzed in this study, this analysis likely underestimates the true frequency of clinically relevant mosaicism in our cohort. As sequencing strategies evolve and directed efforts to detect mosaicism are implemented, an increased contribution of mosaic variants to genetic disease will undoubtedly be uncovered.

## Additional files

Additional file 1: Description of mosaic alternate allele fraction cutoff, and exome sequencing analysis. (DOCX 14 kb)

Additional file 2: **Figure S1.** Correlation of Sanger AAFs with the NGS AAFs of mosaic variants. **Figure S2.** The relationship of the AAF of heterozygous variants to total read depth. **Figure S3.** Estimated AAF for randomly selected 13 mosaic variants. **Figure S4.** Simulation of AAF distribution on SNVs and Indels. **Figure S5.** The distribution of AAF of all heterozygous variants detected in the 900 ES trios. Figure S6 The AAF distribution of the mosaic variants from Tables 1 and 2. (DOCX 392 kb)

Additional file 3: **Table S1.** Summary of mosaic variants and genes according to the inheritance pattern. **Table S2.** Distribution of mosaic mutation types in probands and parents. **Table S3.** Spectrum of different single nucleotide substitutions in proband and parent samples. **Table S4.** Alternate allele fraction of the variants reported in this study. **Table S5.** Mutations spectrum of apparently de novo heterozygous and mosaic autosomal variants in 900 ES trios. (DOCX 38 kb)

Additional file 4 The list of HPO terms of 80 proband phenotypes. (XLSX 16 kb)

## Data Availability

The datasets supporting the conclusions of this article are included within the article and its additional files. Our raw data cannot be submitted to publicly available databases because the patient families were not consented for sharing their raw data, which can potentially identify the individuals.

## Abbreviations

AAF: Alternate allele fraction
AD: Autosomal dominant
AOH: Absence of heterozygosity
AR: Autosomal recessive
ES: Exome sequencing
NGS: Next-generation sequencing
OMIM: Online Mendelian Inheritance in Man
P: Pathogenic
SNV: Single nucleotide variant
UPD: Uniparental disomy
VOUS: Variants of uncertain significance
XL: X-linked
