# Supplementary material for: A clinical survey of mosaic single nucleotide variants in disease-causing genes detected by exome sequencing
Source: Genome Med. 2019 Jul 26;11:48. doi: 10.1186/s13073-019-0658-2 (PMC6660700; doi:10.1186/s13073-019-0658-2)
Supplement: Supplementary file 1 — Description of mosaic alternate allele fraction cutoff, and exome sequencing analysis. (DOCX 14 kb) [file 13073_2019_658_MOESM1_ESM.docx]

**Additional file 1: supplementary Methods**

**Supp Methods**

**Mosaic alternate allele fraction (AAF) cutoff**

In the directly available 900 family trio VCF files, we have identified 4,817 apparently de novo autosomal SNVs that map outside segmental duplications and pseudogenes and have NGS read coverage greater than 50x. We have analyzed distribution of their AAFs and calculated that 95% of AAFs range between 0.36-0.64. Therefore, we use <0.36 or >0.64 for the mosaic AAF cutoff.

**Exome sequencing and analysis**

DRAGEN Pipeline

The output data from the Illumina HiSeq are converted from BCL files to FastQ files according to each sample’s specific adapter sequence using Illumina’s recommended procedure. FastQ data are aligned to the human reference genome using the Edico Dragen Bio-IT Platform. The output of the alignment is a BAM file; QC metrics of the map-align process are recorded for quality review. QC statistics include coverage for exome genes and genes known to be implicated in human disease, mate-pair alignment information as well as number of total and duplicate reads. Variant calling on the BAM file is performed using the Edico Dragen haplotype-based variant calling system and the output is a VCF file.

Mercury Data Analysis Pipeline

The Mercury data analysis pipeline can be accessed at the following website: [www.tinyurl.com/HGSC-Mercury](http://www.tinyurl.com/HGSC-Mercury).

Variant Filtering and Annotation Variant annotation on an exome-wide scale is facilitated by a program developed by the Human Genome Sequencing Center called CASSANDRA, which describes the quality and predicted functional consequences of genomic variants, providing the biological and clinical contexts needed to assess the significance of each variant. Variants are presented to the interpretation team with all quality control metrics produced by the Atlas programs (Atlas-SNP and Atlas-Indel), the sequence data and the theoretical mappability (a measure of sequence degeneracy throughout the region) of the position. AnnoVar (Functional annotation of genetic variants from high-through-put sequencing data; www.openbioinformatics.org) is used to determine a variant’s effect on both a conservative (RefSeq, NCBI Reference Sequence Database) and inclusive (University of California Santa Cruz Genome Browser) gene model set. Multiple databases are applied to assess the variant quality, its minor allele frequency, and disease association.
