# Supplementary material for: A clinical survey of mosaic single nucleotide variants in disease-causing genes detected by exome sequencing
Source: Genome Med. 2019 Jul 26;11:48. doi: 10.1186/s13073-019-0658-2 (PMC6660700; doi:10.1186/s13073-019-0658-2)
Supplement: Supplementary file 2 — Figure S1. Correlation of Sanger AAFs with the NGS AAFs of mosaic variants. Figure S2. The relationship of the AAF of heterozygous variants to total read depth. Figure S3. Estimated AAF for randomly selected 13 mosaic variants. Figure S4. Simulation of AAF distribution on SNVs and Indels. Figure S5. The distribution of AAF of all heterozygous variants detected in the 900 ES trios. Figure S6 The AAF distribution of the mosaic variants from Tables 1 and 2. (DOCX 392 kb) [file 13073_2019_658_MOESM2_ESM.docx]

**Additional file 2: supplementary Figures**

Fig S1 Correlation of Sanger AAFs with the NGS AAFs of mosaic variants


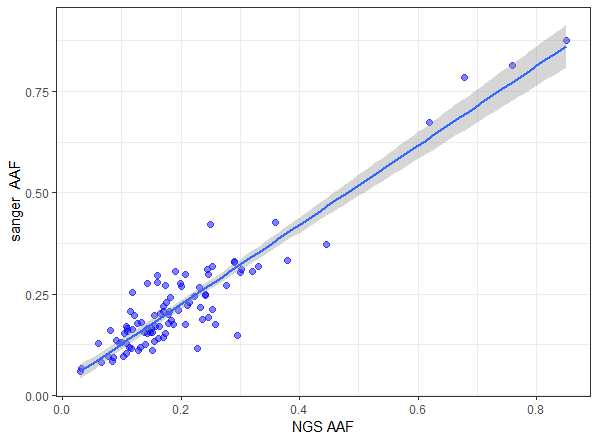


Fig S2 The relationship between the AAF of heterozygous variants detected in the 900 ES trios and their read depth


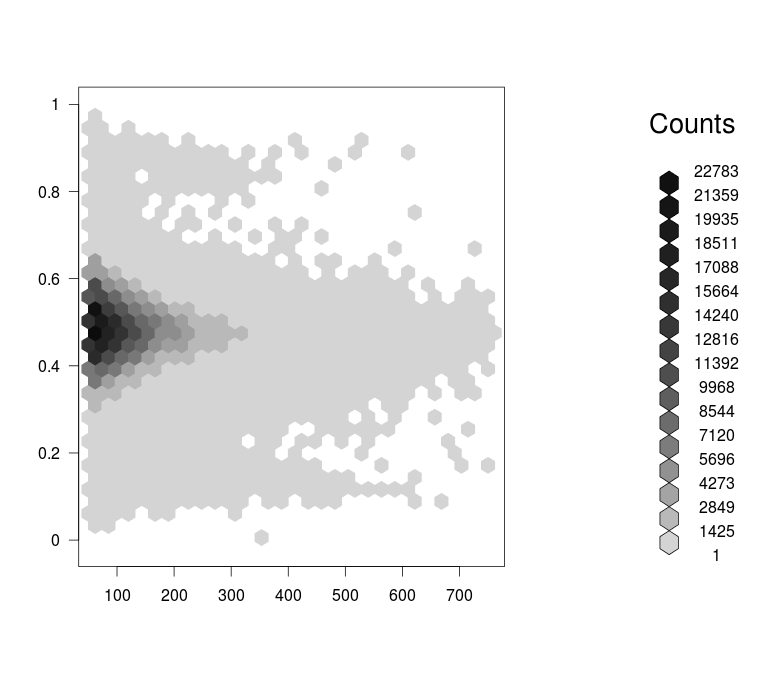


Fig S3 Estimated AAF for randomly selected 13 mosaic variants in our study


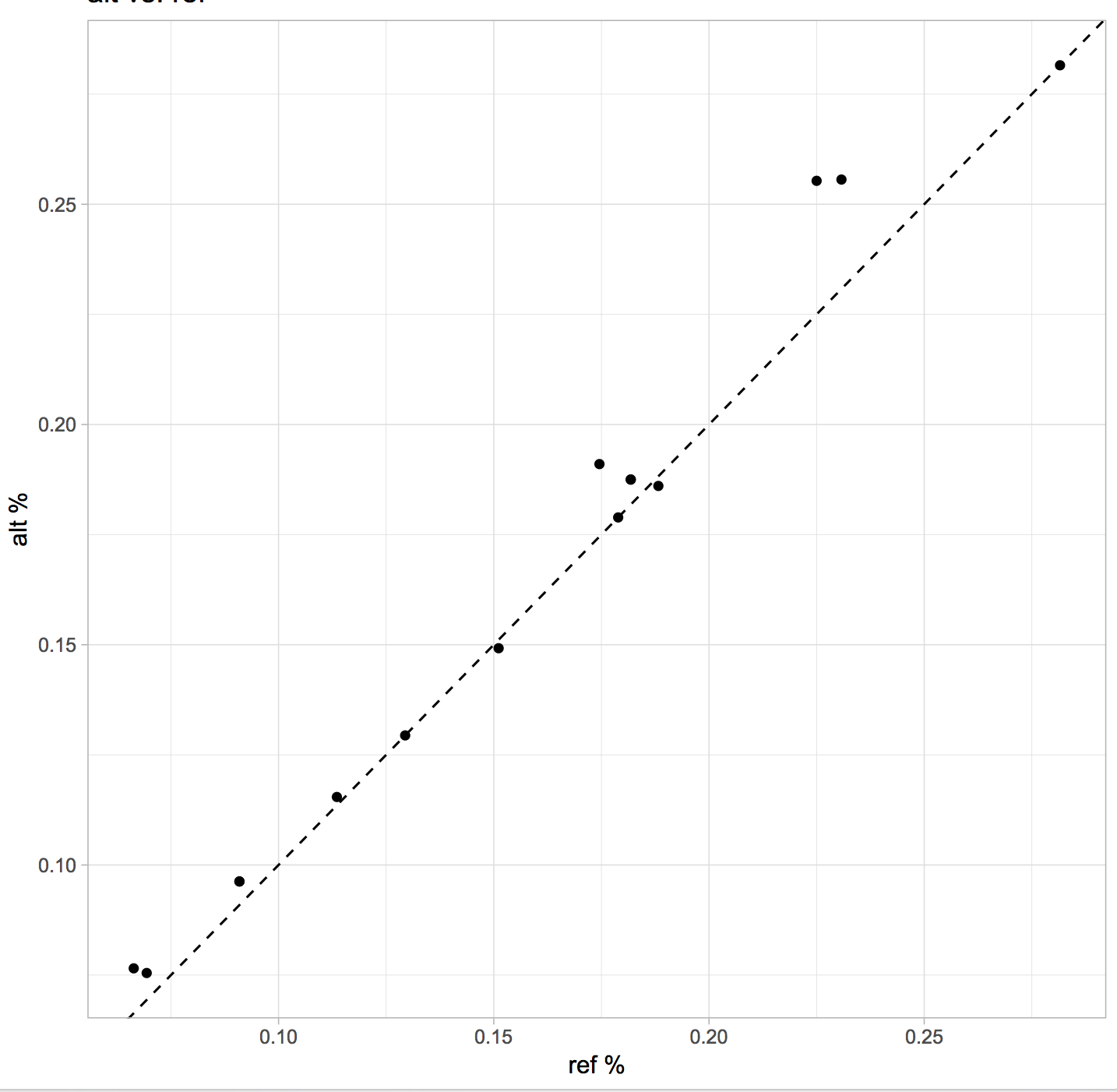


Reference hg19 chromosomes were downloaded from UCSC (hgdownload.cse.ucsc.edu), and individual bases in the reference chromosomes were altered to match the minor allele using the personal genome constructor (vcf2diploid, [https://github.com/abyzovlab/vcf2diploid)](https://urldefense.proofpoint.com/v2/url?u=https-3A__github.com_abyzovlab_vcf2diploid-29&d=DwMGaQ&c=ZQs-KZ8oxEw0p81sqgiaRA&r=mteXiaglztWWFcxh2-kOdw&m=WeX8fXnQdt2QKYemsb5GadSwKOUjuI9orMsn99Z5iVU&s=3zK7CKkzGcFJX9O3g7PQfTzpW6WHYVVpPzU5WgYqK88&e=). The reference (“ref”) and modified (“alt”) chromosomes were used for alignment.  FastQ data were obtained from ordinary demultiplexing procedure or when not available by reversing existing BAM file to create FastQ.  The FastQ files raw data were then used to test mapping bias by alignment using STAR 2.6.1d. For tabulation of coverage, analysis was restricted to properly aligned pairs where reads were filtered for mapping quality (mapQ > 30). The AAF for both the “ref” aligned results and the personal genome results were tabulated as the Alt read depth over the total depth. The findings suggest comparable VAF for the mosaic samples using both the modified personal genome reference and the human reference, but with a slight under-estimation for the reference aligned results.

Fig S4 Simulation of AAF distribution on SNVs and Indels

A


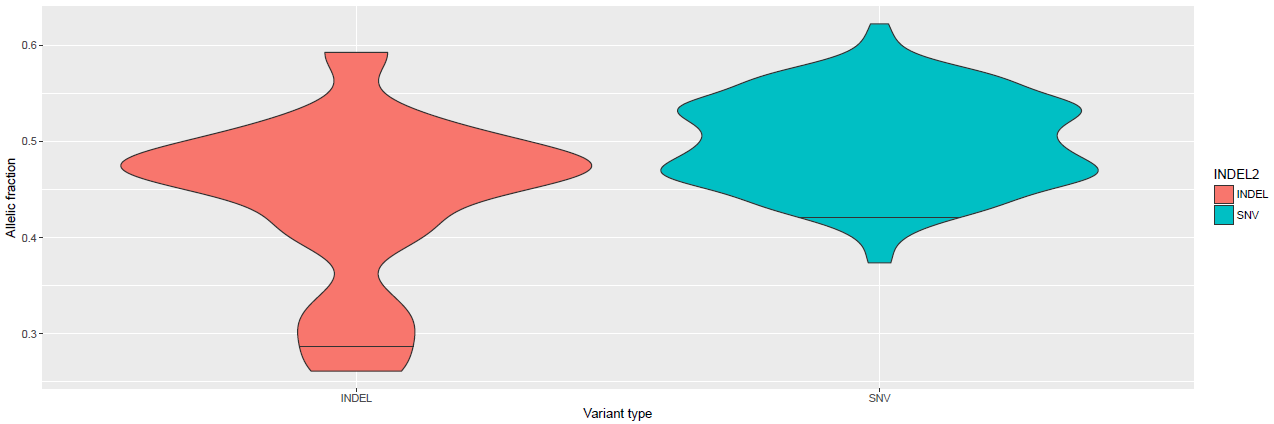


B


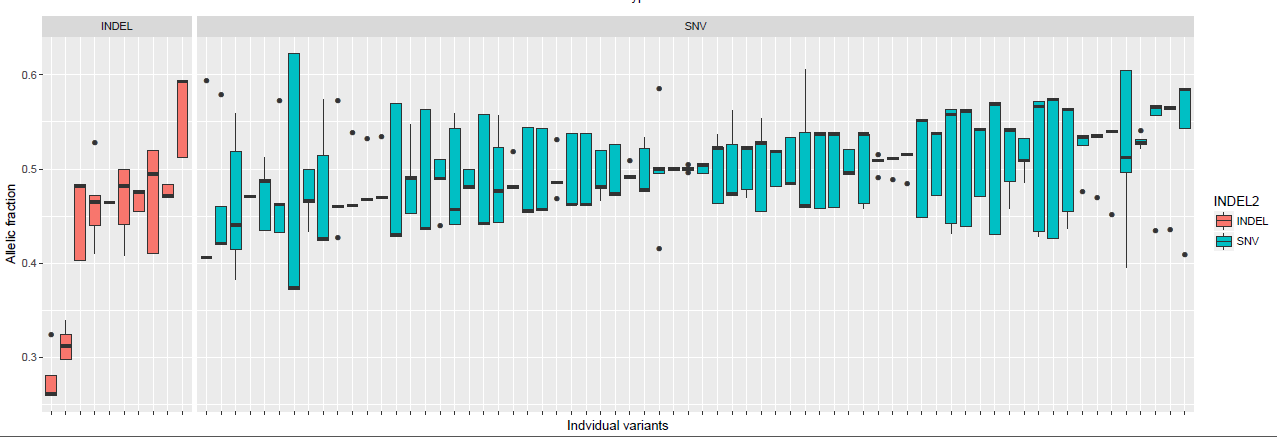


To evaluate the effect of the potential alignment bias on AAF, we have performed the following simulation experiment using VarSim and ART softwares (1-3) For each of the selected 78 mosaic variants (including 68 SNVs and 10 indels), we have simulated the sequencing reads for the fragment of reference genome (GRCh37) that includes a variant and 2 kb flanking regions with the average coverage of 100x. The variants were simulated as regular heterozygous mutations with expected AAF of approximately 50%. Reads from FASTQ files generated in the previous step were then mapped to the reference genome using bwa_mem (4). Finally, we have collected the pileup information for each variant position and computed AAF using a custom R script. To assess the variability of AAF for a given variant, we have repeated the entire procedure 100 times. Distribution of AAF differ significantly between indels and SNVs (Additional file2: Figure S4B). The horizontal black lines on the violin plots indicate the fifth percentile of AAF (0.426 and 0.27 in case of SNVs and indels, respectively). We also observe the variability of AAF distribution among individual variants (Additional file2: Figure S4B). Importantly, only 2 out of 78 variants (both are indels) have average AAF below 0.36.

Fig S5 The AAF distribution of all heterozygous variants detected in the 900 ES trios


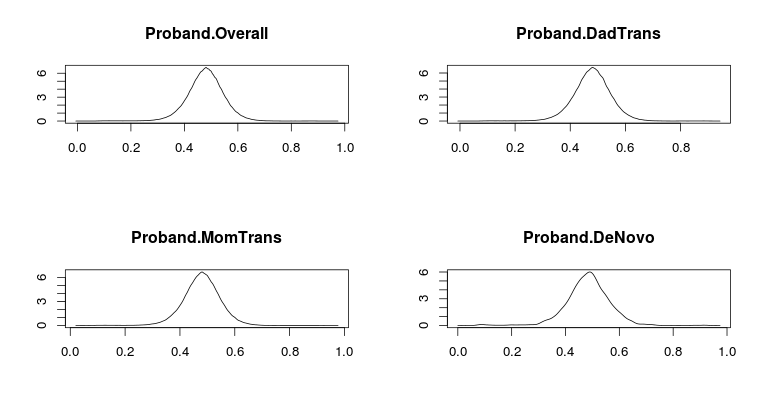


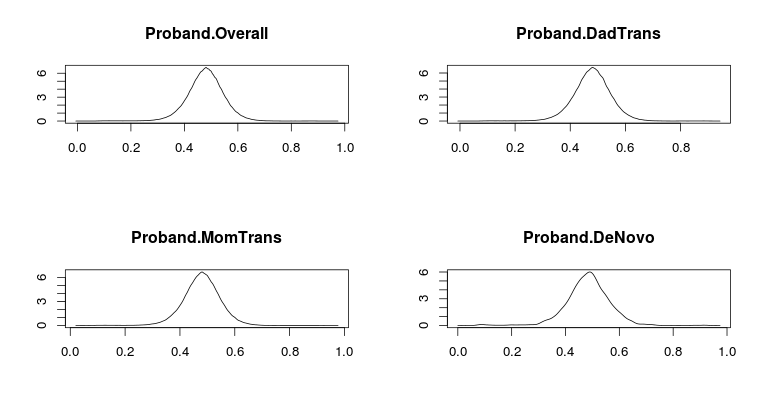


Left: AAF distribution of apparently *de novo* heterzgyous variants

Right: overall AAF distribution of heterzgyous variants

Fig S6 The AAF distribution of the mosaic variants from Table 1 and 2


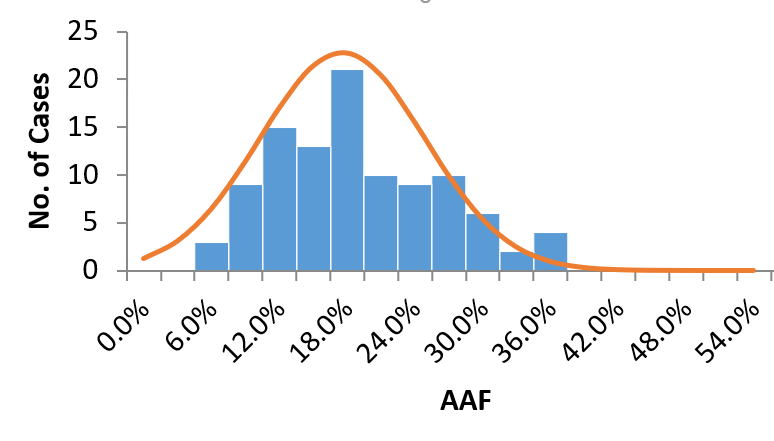


*X-linked mosaic variants in the males were excluded form this distribution.

**Supplementary References:**

1. Mu JC, Mohiyuddin M, Li J, Bani Asadi N, Gerstein MB, Abyzov A, et al. VarSim: a high-fidelity simulation and validation framework for high-throughput genome sequencing with cancer applications. Bioinformatics (Oxford, England). 2015;31:1469-71.

2. Huang W, Li L, Myers JR, Marth GT. ART: a next-generation sequencing read simulator. Bioinformatics (Oxford, England). 2012;28:593-4.

3. Escalona M, Rocha S, Posada D. A comparison of tools for the simulation of genomic next-generation sequencing data. Nat Rev Genet. 2016;17:459-69.

4.Li H. Aligning sequence reads, clone sequences and assembly contigs with BWA-MEM. arXiv:13033997v2 [q-bioGN]. 2013.
