# Supplementary material for: A clinical survey of mosaic single nucleotide variants in disease-causing genes detected by exome sequencing
Source: Genome Med. 2019 Jul 26;11:48. doi: 10.1186/s13073-019-0658-2 (PMC6660700; doi:10.1186/s13073-019-0658-2)
Supplement: Supplementary file 3 — Table S1. Summary of mosaic variants and genes according to the inheritance pattern. Table S2. Distribution of mosaic mutation types in probands and parents. Table S3. Spectrum of different single nucleotide substitutions in proband and parent samples. Table S4. Alternate allele fraction of the variants reported in this study. Table S5. Mutations spectrum of apparently de novo heterozygous and mosaic autosomal variants in 900 ES trios. (DOCX 38 kb) [file 13073_2019_658_MOESM3_ESM.docx]

**Additional file 3: supplementary Tables**

**Table S1:** Summary of mosaic variants and genes according to the inheritance pattern

| **Inheritance pattern** | **No. of genes** | | **No. of variants** | |
| --- | --- | --- | --- | --- |
| AD, AD/AR | 60 | 56.1% | 67 | 55.8% |
| X-linked | 31 | 29.0% | 33 | 27.5% |
| AR | 8 | 7.5% | 8 | 6.7% |
| AD/somatic | 6 | 5.6% | 10 | 8.3% |
| Somatic | 2 | 1.9% | 2 | 1.7% |
| Total | 107 |  | 120 |  |

**Table S2:** Distribution of mosaic mutation types in proband and parents

| Variant type | Total | Proband | Parents |
| --- | --- | --- | --- |
| Total | 120 | 80 | 40 |
| Missense | 81 | 55 | 26 |
| Nonsense | 17 | 11 | 6 |
| Splicing site | 6 | 2 | 4 |
| Frameshift | 16 | 12 | 4 |
| CpG site | 35/104 | 24/68 | 11/36 |

**Table S3:** Spectrum of different single nucleotide substitutions in proband and parent samples

|  | **Proband** | **parents** |
| --- | --- | --- |
| C>T | 20 | 10 |
| C>A | 1 | 2 |
| C>G | 1 | 0 |
| A>T | 5 | 1 |
| A>C | 0 | 2 |
| A>G | 4 | 0 |
| T>C | 2 | 2 |
| T>A | 3 | 0 |
| T>G | 2 | 2 |
| G>C | 3 | 6 |
| G>A | 21 | 7 |
| G>T | 6 | 4 |
| Total | 68 | 36 |

**Table S4:** Alternate allele fraction of the variants reported in this study

|  | | **Proband** | | **Parental** | |
| --- | --- | --- | --- | --- | --- |
| **Inheritance pattern** | | No. of cases | Average VAF% | No. of cases | Average VAF% |
| AD, AD/AR | | 39 | 19.6 ± 6.6 | 27 | 16.1 ± 8.2 |
| X-linked | Female | 9 | 15.8 ± 3.3 | 8 | 8.6 ± 3.5 |
|  | Male | 15 | 32.6 ± 24.4 | 1 | 67.8 |
| AR | | 6 | 27.7 ± 23.5 | 2 | 16.1 |
| AD/somatic | | 9 | 20.8 ± 6.0 | 1 | 20.4 |
| Somatic | | 2 | 22.1 | 0 | N/A |
| Total | | 80 |  | 39 |  |

The grandparent case was not included.

**Table S5:** Mutations spectrum of apparently de novo heterozygous and mosaic autosomal variants in 900 ES trios

|  | No. of SNVs with AAF >0.36 |  | No. of SNVs with AAF <0.36 |  |
| --- | --- | --- | --- | --- |
| C>T | 996 | 20.7% | 48 | 20.1% |
| C>A | 203 | 4.2% | 14 | 5.9% |
| C>G | 272 | 5.6% | 14 | 5.9% |
| A>T | 117 | 2.4% | 6 | 2.5% |
| A>C | 173 | 3.6% | 13 | 5.4% |
| A>G | 715 | 14.8% | 41 | 17.2% |
| T>C | 622 | 12.9% | 38 | 15.9% |
| T>A | 105 | 2.2% | 7 | 2.9% |
| T>G | 166 | 3.4% | 6 | 2.5% |
| G>C | 237 | 4.9% | 9 | 3.8% |
| G>A | 999 | 20.7% | 34 | 14.2% |
| G>T | 212 | 4.4% | 9 | 3.8% |
|  | 4817 |  | 239 |  |
